# Antibody Affinity Maturation in Fishes—Our Current Understanding

**DOI:** 10.3390/biology4030512

**Published:** 2015-07-31

**Authors:** Brad G. Magor

**Affiliations:** Department of Biological Sciences, University of Alberta, Edmonton, AB T6G-2E5, Canada; E-Mail: bmagor@ualberta.ca; Tel.: +1-780-492-5956; Fax: +1-780-492-9234

**Keywords:** fishes, antibody, affinity maturation, germinal center, somatic hypermutation, activation-induced cytidine deaminase, AID, Aicda, evolution

## Abstract

It has long been believed that fish lack antibody affinity maturation, in part because they were thought to lack germinal centers. Recent research done on sharks and bony fishes indicates that these early vertebrates are able to affinity mature their antibodies. This article reviews the functionality of the fish homologue of the immunoglobulin (Ig) mutator enzyme activation-induced cytidine deaminase (AID). We also consider the protein and molecular evidence for Ig somatic hypermutation and antibody affinity maturation. In the context of recent evidence for a putative proto-germinal center in fishes we propose some possible reasons that observed affinity maturation in fishes often seems lacking and propose future work that might shed further light on this process in fishes.

## 1. Introduction

In the mammalian paradigm of antibody affinity maturation a few activated B-cells (plasmablasts) and T_H_-cells are sequestered to primary lymphoid follicles where the former cells (now centroblasts) proliferate and acquire “random” point mutations in their VDJ exons. These newly modified daughter cells (centrocytes) then compete for limited antigen, trapped in complex with antibody or complement, on the surface of follicular dendritic cells (FDCs). Successful competitors are rescued from pre-programmed apoptosis by T_H_-cells and then further clonally expand before differentiating into either plasma or memory B-cells. The locally expanded population of centroblasts and centrocytes generates the histologically distinct germinal centers that will eventually dissipate as the infection is resolved. The end result of this process is an expanded B-cell population expressing antibodies with as much as a 1000-fold higher affinity for their cognate antigen than that of the original parental cell that first nucleated the primary follicle.

Though all gnathostomes have B-cells and develop memory responses to vaccines there has been considerable debate on whether ectothermic vertebrates have a complete antibody affinity maturation process. This debate has centered on the apparent absence of two elements in the mammalian system—histologically distinct germinal centers and the development of a dominant pool of high affinity circulating antibodies following immunization.

**Figure 1:**
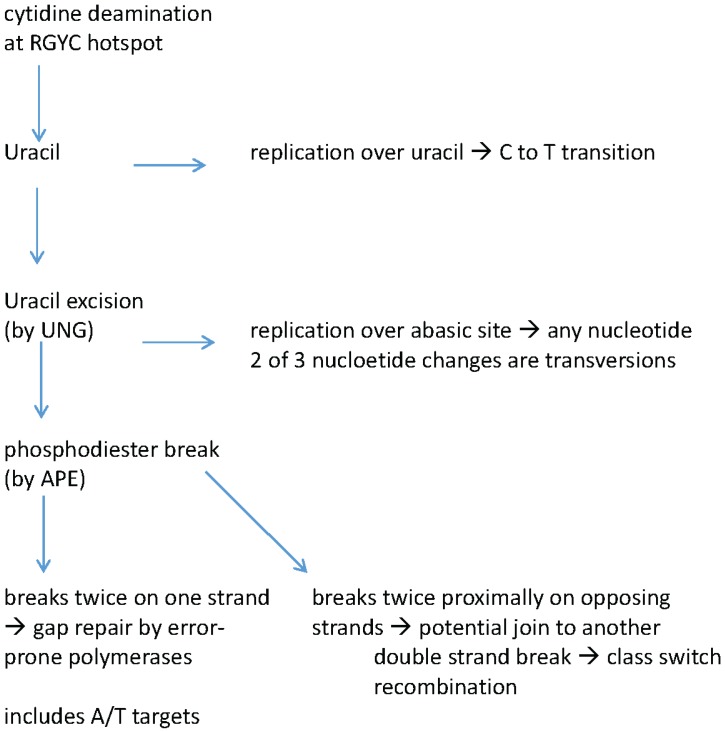
Schematic of outcomes of activation induced cytidine deaminase (AID) mediated cytidine deaminations in immunoglobulin genes. AID targets cytidines within the RGYW hotspot motif. UNG—Uracil-DNA-glycosylase; APE—apurinic/apyrimidinic endonuclease.

Though the patterns of mutation occurring in Ig genes had been previously noted, the identification of the Ig mutator enzyme, activation-induced cytidine deaminase (AID or Aicda) has clarified how specific mutation patterns arise. To briefly summarize the mutational process (reviewed in [1]), the first step is transcription through the VDJ exon. AID binds ssDNA within the transcription bubble where it can deaminate cytidines, generating a uracil in the process (Figure 1). The cytidines that are targeted are typically within the context of the mutational hotspot motif RGYW (and its compliment YCRW; where Y is a pyrimidine, R is a purine and W is weak or A/T). Replication over uracils results in a C to T transition at the original site while removal of the uracil in dsDNA by uracil-DNA-glycosylase (UNG) has a number of possible outcomes which are summarized in Figure 1. Replication over an abasic site leads to any base occupying the site of the original cytidine. Of the replacement nucleotides two would represent pyrimidine to purine transversions (C to A or G). Alternatively the abasic site may have its phosphodiester backbone cleaved by apurinic/apyrimidinic endonuclease (APE). Strand breaks on opposing strands is thought to necessarily precede class switch recombination. Strand breaks on the same strand can lead to strand gaps that are filled in by polymerases that, in this context, are error prone and introduce mutations at A-T nucleotides as well.

The characterization of the mutation processes initiated by AID, and technological advances in protein chemistry and sequencing have provided a clearer understanding of how affinity maturation operates in both homeotherms and ectothermic vertebrates, and it is these advances that we will consider in greater detail.

## 2. Earlier Research—Fishes Have Somatic Hypermutation of Ig Genes

The large number of V_H_- and V_L_-elements in the genomes of all vertebrates, as revealed by southern blots, made it difficult for earlier researchers to assess whether apparent point mutations were derived in somatic cells or represented a hitherto uncharacterized variable element. Many of the problems associated with this conundrum were overcome in two ectothermic vertebrate systems, the Chondrichthyes and African clawed toad. Louis Du Pasquier [2] and colleagues used isogenic (and homozygous at the IgH allele) *Xenopus* to determine the dominant V_H_-element encoding the antibodies of B-cells that could recognize haptenated keyhole limpet hemocyanin (DNP-KLH). Among the many seminal observations made in these DNP-KLH hyperimmunized toads was: (1) there were mutations accumulating in somatic B-cells, at a rate estimated to be 4- to 7-fold lower than that in mammalian Ig genes; (2) Increases in antibody affinity for DNP were only 5- to 10-fold 4 weeks after immunization; (3) Unlike mammals there was a strong GC bias for sites of mutation—particularly in codons for serine (RGY/YCR); (4) There was evidence for clonal lineage accumulation of mutations; (5) The ratio of replacement to silent mutations (R/S) was not statistically above the rate expected for random (non-selected) mutations in the complementarity determining regions (CDRs), which suggested to them that there was not a germinal center-like selection process occurring. These studies in amphibians of course did not necessarily predict what might be occurring in the earlier divergent fishes, though the discovery in the 1990’s of a new Ig isotype (IgNAR) in sharks and rays, with a very limited number of V-elements did open the way for determining that Ig somatic hypermutation did exist before the divergence of bony fishes. The Ig loci in sharks and rays are distinct in that there are hundreds of loci, of multiple isotypes, that have only one or a few V-, D- and J-elements per locus. In the case of the IgNAR (new antigen receptor) there is only a few loci with a single V_NAR_-element each and only two loci appear to be expressed—at least in adult nurse sharks [3]. Using cDNA obtained from wild adult nurse sharks Flajnik and colleagues characterized over a thousand somatic mutations in IgNAR transcripts. Unlike the amphibian mutations there was no GC bias in targeting though serine codons were mutational hotspots [4,5]. Furthermore there was evidence of antigen driven selection of B-cells that had mutations predominating in the complementarity determining regions of the VDJ exons. Because the sharks had not been immunized it was not determined whether these seemingly antigen-selected clones generated antibodies of much higher affinity. As was the case for *Xenopus* past immunizations of sharks and rays generated only modest affinity increases in IgM responses to antigen [6,7]. The existence of an effective somatic mutation system in sharks and amphibians meant it likely that it had also been retained in fishes.

While fishes (and other ectothermic vertebrates) lack a histochemically distinct germinal center (by hematoxylin and eosin or by peanut agglutinin binding) there had been evidence for a region in which antigen would be retained in lymphoid tissues for periods of more than 3 months. The sites of antigen retention are the ellipsoid sheaths and adjacent melanomacrophage centers which are populated by numerous macrophages that have accumulated pigments (melanin, lipofuscin and hemosiderin) and are distinguishable histologically. Several authors have noted that antigen [8,9,10] perhaps in complex with antibody [11] are retained on or proximal to these cells. This, along with the presence of B-cells in or proximal to the clusters [12,13] had led to the hypothesis that these clusters are acting as or in an analogous manner to lymphoid follicles or germinal centers. If this were true then an important distinction between melanomacrophage clusters and germinal centers is that in the latter the amount of antigen retained on the follicular dendritic cells and/or the number of antigen retaining cells appears to be far less then for melanomacrophage clusters—at least following vaccination. 

## 3. Recent Work on Teleost Fish

### 3.1. Antibody Affinity Measurements

Two groups have looked at development of antibody affinity in teleost's following vaccination. Rainbow trout given 200 μg of FITC-KLH and an identical booster at 4 weeks developed a 3-fold increase in total serum antibody affinity (measured as K_d_ by BIAcore) by 14 weeks [14]. This work did not resolve whether the increased affinity was due to new Ig variants arising from somatic mutation and selection, or just selection for pre-existing B-cells expressing high affinity antibody for the priming antigen. This latter issue was addressed by Steve Kaattari’s group who used an affinity-partitioning ELISA system to classify antibodies into different affinity groups and track their development with time following immunization [15]. The methodology is not laid out in their earlier papers but a 2010 paper does describe an approach that seems consistent with earlier publications: 200 to 300 g rainbow trout are immunized once i.p. with 100 μg of TNP-KLH and antibody affinities were monitored for 30 weeks [16]. The antibody affinity increases observed in these papers was modest (3 to 10-fold affinity increase) though importantly they noted that the higher affinity antibody populations first appear later in the humoral immune response suggesting that these were the products of Ig variants arising from an affinity maturation process. An observation, with as of yet unknown significance, was that concomitant with increased antibody affinity in rainbow trout was an increase in the proportion of IgM molecules that exist as tetramers [15,16,17]. As the authors note studies in at least 15 fish species have observed that serum IgM can exist as monomers, dimers, trimers or tetramers. These studies noted a correlation between binding affinity and polymerization with higher affinity antibodies being frequently found in tetrameric form. Certainly one advantage in being multivalent is an increase in overall avidity of the molecule, even if individual variable region binding affinity is low. Besides increased avidity, it appears that the tetrameric IgM has a longer half-life than less polymerized forms when injected back into the fish [16]. How binding an antigen with high affinity selectively increases polymerization, or other advantages of increased polymerization during humoral immune responses remains to be determined.

### 3.2. Fish Have a Fully Functional Ig Mutator Enzyme—AID

Activation induced cytidine deaminase (AID or Aicda) was first identified in 1999 [18] and was quickly established as the initiator of both Ig somatic hypermutation and class switch recombination [19,20]. Mechanistically (reviewed in [21]) AID targets cytosines on ssDNA in the transcription bubble where deamination generates a uracil. Replication over the uracil leads to a C to T transition. However uracil N-glycosylase (UNG) may remove the uridine leaving an abasic site, replication over which leads to any base at that position in the new strand (*i.e.*, transitions and transversions). Alternatively the abasic site may be targeted by apurinic/apymidinic endonuclease (APE), which cleaves the phosphodiester backbone causing a strand break. Strand breaks on the same strand can lead to excision of a segment of DNA that then is filled in by “error-prone” polymerases (likely Pol η) leading to mutations at A:T. Strand breaks on opposing strands at heavy chain switch sites precedes class switch recombination, the canonical form of which is lacking in fish. The absence of class switching in fish may relate more to an absence of an appropriately organized Ig gene, rather than the absence of elements necessary to mediate the process. In tetrapods that class switch, appropriate Ig organization is the translocon Ig locus in which there are several constant domain exon clusters preceded by cryptic promoters and switch repeat sequences. However, even this organization is not absolutely necessary for switching as sharks have recently been found to have a somewhat novel form of class switching. Sharks have multi-cluster Ig genes. These Ig genes are somewhat simplified Ig loci, typically with one or a few V-, (D-) and J-elements and a single group of constant domain encoding exons. There are multiple such loci within chromosomal regions and, by virtue of locus specific nucleotide patterns, Ellen Hsu and colleagues have determined that VDJ exons of one locus can be switched to the constant domain exons of another locus [22,23]. There is no evidence of switch regions or cryptic promoters in the shark Ig loci, though switching is able to take place and appears commensurate with AID expression. The functional advantages of class switching in sharks have not yet been established.

The first fish homologue of AID was reported in 2004 [24] and came from channel catfish. Subsequent to that a number of other fish AID homologues have been reported [25] or their sequences deposited in Genbank. The phylogeny and many functions of fish AID have been examined in detail in a recent review [26]. Among the key observations are that the AID of fugu, zebrafish and channel catfish, when expressed in bacteria, yeast or mouse cell lines can be potent inducers of mutation, though in all cases zebrafish AID was a more potent mutator than that of fugu or catfish [27,28]. Also, though teleosts do not have classical class switch recombination, all three teleost AIDs could drive class switching in mouse AID^−/−^ B-cells [27,28]. Both groups noted some temperature sensitivities of the fish AIDs in these systems and it has since been determined that a single amino acid difference, common to fugu and catfish, substantially diminishes their ability to bind ssDNA at higher temperatures, but not at the temperatures of their native environments [29]. We had previously observed that AID expression could be induced in a channel catfish B-cell line (1B10; [30]) and concomitant with this activation there was accumulation of somatic mutations in the endogenous IgH VDJ exon [29,30].

To the extent that it has been investigated, the regulation of fish AID expression [24,30,31,32], activation [33] and intracellular trafficking [34,35] are all evolutionarily conserved.

### 3.3. Somatic Hypermutation Patterns in Fish Immunoglobulins

A hallmark of germinal center based affinity maturation is that, within each germinal center a clonal lineage of B-cells will develop (characterized by unique common V(D)J recombination sequences) that independently accumulate new mutations within the VDJ exon and pass these onto daughter cells that add to the mutations inherited from the parental cell [36]. Several investigations of mutational patterns in fish Ig genes have revealed evidence for such lineage development, though not all believe they are due to antibody affinity maturation. Lobb and colleagues [37] made a μ-chain specific cDNA library from the spleen of a vaccinated channel catfish. From 187 clones and 48 kb of sequence that matched know V_H_ or J_H_ segments they identified 459 mismatches. The majority of mismatches were transitions at G:C within the RGYW hotspot motif (Table 1). Furthermore, many of the mutations at adenines also occurred within this motif. Their analysis of the distribution of replacement *vs.* silent mutations in the framework and complementarity determining regions led them to conclude that there was no evidence for antigen-selection of B-cells harboring the altered antibodies and though they did observe evidence of clonal lineage development, they intimated that the mutations were generated as part of the development of the primary antibody repertoire [37].

**Table 1:** The nature and spectrum of somatic mutations in cartilaginous and bony fishes.

Species	Gene Analyzed	# & Types of Substitutions	Nucleotide Bias ^a^	% That are Transitions	% in RGYW Hotspot ^b^	Lineage Development	Reference
channel catfish	V_H_	459	58% GC	60	47	Yes	[37]
zebrafish	V_L_	93	59% GC^c^	85	49	Yes	[38]
nurse shark	V_H_	78 tandem	56% AT	36	39	No	[39]
nurse shark	V_H_	53 singlet	57% GC	53	39	No	[39]
nurse shark	V_L_	338 tandem	No	38	42	Yes	[40]
nurse shark	V_L_	293 singlet	No	58	43	Yes	[40]
nurse shark	V_L_	245 tandem ^d^	59% AT	31	46	?	[41]
nurse shark	V_L_	187 singlet ^d^	No	55	46	?	[39]
nurse shark	V_NAR_	231 synonymous	No	62	?	?	[5]
nurse shark	V_NAR_	523	No	39	?	?	[3,42]

^a^ values are not necessarily corrected for the proportion of nucleotides in the target sequence. ^b^ RGYW hotspot makes up 20% to 30% of the sequence analyzed in each of these species. ^c^ significant G bias. ^d^ our calculations, based on data presented—excludes tandem or triplet substitutions. ^e^ substitution in nucleotides that would not be under selection—*i.e.*, non-coding.

Expansion of antibody primary repertoire through somatic mutations does occur in several mammals and all birds studied to date [43,44,45,46]. Though there is evidence that sharks diversify their TcR γ- and α-chain repertoires by post-rearrangement somatic mutations [47,48]; M. Criscitiello Pers. Comm.), there is no evidence that fish or sharks diversify their Ig primary repertoire in a similar fashion. A recent high-throughput sequence analysis of the Ig repertoires of 51 zebrafish through development found very few mutations in young fish immunoglobulins [49]. However, they found that the acquisition of somatic mutations increases with age and they interpreted this to correlate with degree of past pathogen exposure [49]. These authors did not analyze the nature of the mutations (e.g., replacement *vs.* silent or framework *vs.* CDR).

The only other analyses of *in vivo* generated somatic mutations (in teleost’s) have been done in zebrafish. Marianes and Zimmerman [38] made an enriched cDNA library for a single V_L_ locus from a mature non-vaccinated zebrafish. As with the catfish there was preponderance of transition mutations at G:C within the canonical hotspot motif RGYW (Table 1). As these authors suggest the high rate of transition mutations and paucity of A:T mutations could be an indication that UNG targeting of uracils occurs at a lower frequency in teleosts. If this is the case it would seem to represent a loss of process as analyses of shark Ig mutations indicate a high degree of A:T targeting and nucleotide transversion, though this is variable among studies and specific Ig genes analyzed (Table 1).

The best molecular evidence to date for affinity maturation in early gnathostomes comes from the nurse shark IgNAR. Because this antibody is derived from a single gene (there is no light chain) it is possible to build phage display libraries that tie the expressed protein to the gene encoding it. Flajnik and colleagues hyperimmunized a nurse shark with HEL (hen egg lysozyme) and then panned for phage generated from IgNAR libraries from blood and lymphoid organs [50]. Starting with these IgNAR sequences they were able to obtain lineages of IgNAR gene (encoding anti-HEL antibodies) that had successive mutations. When tested for binding affinity (by BIAcore), it was found that increases in (some) acquired mutations correlated with increases in binding affinities—which is what would be expected for an affinity maturation process.

The IgNAR like other shark isotypes have some mutational patterns that distinguishes them from bony fishes. As stated above there is equal mutation of A:T and G:C as well as some additional non-canonical mutational hotspots [39,40,41]. Furthermore many of the mutations are found in tandem on adjacent nucleotides (Table 1), which is a feature that thus far is only known to be prevalent in shark Ig mutations.

### 3.4. A Cellular Context for Generation of Somatic Hypermutations

**Figure 2:**
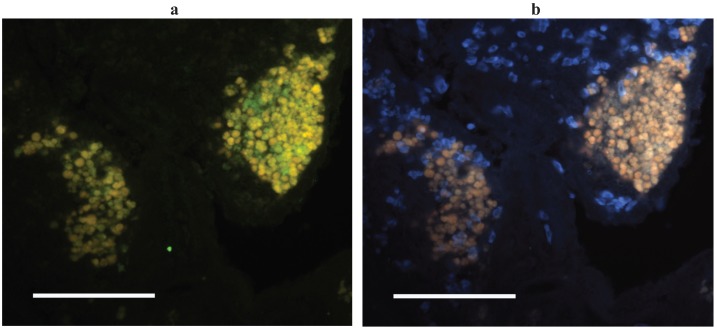
AID expressing cells in channel catfish spleen. (**a**) *In situ* hybridization with AID anti-sense probe (green) under excitation for FITC. Melanomacrophages autofluoresce yellow; (**b**) UV excitation elicits orange/brown autofluorescence of only melanomacrophages (and RBCs in blue). Methods are as described in [28].

Though ectothermic vertebrates lack a histologically distinguishable germinal center a number of studies on affinity maturation in human autoimmune disorders had noted that loose and often ectopic aggregates of B- and T-cells, with or without associated follicular dendritic cells, could generate antigen selected B-cells [51,52,53]. With this in mind and with the catfish AID as a marker of sites of somatic hypermutation we set out to identify the tissue architecture associated with affinity maturation in channel catfish tissues. Two approaches were taken: laser capture microdissection of cell subsets from histological sections, and *in situ* hybridization (ISH) on histological sections using anti-sense probes for IgH or AID. As a starting point for the microdissection we isolated leukocyte rich regions and autofluorescent melanomacrophage clusters and were somewhat surprised to find that AID was expressed solely in the melanomacrophage clusters, while IgH (μ-chain), TcRβ and CD4 were expressed in both tissue subsets [30]. This finding was corroborated by our AID ISH which showed that AID expressing cells were always proximal to melanomacrophages (in spleen, kidney and intestine) though melanomacrophages clusters did not always have associated AID expressing cells (all liver melanomacrophage clusters and occasional clusters in other tissues) [30] (Figure 2). As noted above, melanomacrophage clusters had previously been suggested as a primordial germinal center by virtue of their retention of antigen for long periods of time following immunization [8,9,10,11]. This antigen retention, which can be considerable, and may also occur in the ellipsoid sheath that is proximal to the clusters [9,54], conceivably functions in a manner analogous to the antigen retained on FDCs. While antigen retention has been attributed to the melanomacrophages, these cells are surrounded by a reticular cell stroma that can be quite extensive [55,56,57]. Reticular cells would also seem to be a candidate for antigen trapping by virtue of their lineage relationship to follicular dendritic cells [58].

This cellular context for somatic hypermutation is only known for a single fish species and we should note that similar AID ISH done in the spleen of *Xenopus* did not reveal clusters of AID^+^ cells [59].

### 3.5. Why is Affinity Maturation so Poor in Fish?

While much needs to be done before being able to conclude that melanomacrophage clusters are primordial germinal centers (see below). Presuming for now that they are, what then limits affinity maturation? We imagine two possible scenarios one of which is an artifact of how we vaccinate fish. In mammalian germinal centers the ratio of centrocytes to FDCs appears such that there is only limited trapped antigen available for which emerging centrocytes have to compete. This then will favor selection of B-cells expressing the highest (among competitors) affinity antibodies for the antigen. In melanomacrophage clusters the AID expressing cells are far outnumbered by antigen-trapping cells, be they melanomacrophages or reticular cells. As such an oversupply of trapped antigen will ensure that any B-cell with antibody that is still capable of binding antigen will be selected for survival and clonal expansion to memory and plasma cells. While this scenario proves confounding for those trying to unravel the affinity maturation process, it might be quite advantageous to smaller animals trying to quickly resolve an infection. Moreover, as has been noted by many before, the high valency nature of IgM means that it need not necessarily have high affinity in order to have strong binding avidity to a pathogen.

The second scenario which is not necessarily exclusive of the first, is that the kinetics of activated B-cell proliferation is so slow that at any given time there are relatively few centrocytes competing for antigen—again resulting in the selection of all B-cells still capable of binding the antigen.

### 3.6. Issues to Resolve around Antibody Affinity Maturation in Fishes

The autofluorescent nature of the pigments retained in melanomacrophages has been both helpful in that it allows for their identification without staining, and problematic insofar as the pigments quench any underlying fluorescence by other sources such as immunofluorescent cell labels [30].

We recently published on the observation that melanomacrophage clusters in cyprinids frequently are extensively encapsulated by reticular cells and this in turn makes them relatively easy to isolate [57].

(1) If the melanomacrophage clusters are functioning in a manner analogous to germinal centers then the B-cells contained within clusters should represent clonal lineages derived from a few nucleating activated B-cells. This is the case in mammalian germinal centers and really is the gold standard for any form of germinal center. We have preliminary evidence (manual sequencing of μ-chain cDNA libraries from individual clusters) to suggest this will be the case catfish and goldfish, but to resolve it fully will require next generation sequencing of the majority of IgH transcripts in individual clusters.

(2) The kinetics of cell proliferation and apoptotic cell death in melanomacrophage clusters needs to be revisited using laser scanning confocal microscopy. By this approach, fluorescence signals quenched by melanomacrophage pigments can be revealed and labeled cells correctly enumerated [28].

(3) Determining which cell type (melanomacrophage *vs.* reticular cell) traps antigen would allow for the determination of which types of receptors facilitate trapping (complement or Fc receptors) and chemokines (e.g., CXCL13) used for the recruitment of activated B- and T_H_-cells.

## 4. Conclusions

Over the last 20 years it has been established that several features consistent with antibody affinity maturation were in place in early gnathostomes.

1) In both fish and elasmobranches there is accumulation of somatic point mutations in V(D)J exons with preferential targeting of the canonical hotspot motif RGYW.
2) There is evidence for development of mutation lineages consistent with a hypermutation process occurring in proliferating B-cells.
3) There are some clear differences in how mutations in the fish and elasmobranches are resolved. Fish have limited mutations in A:T while in sharks there is evidence for gap repair by error prone polymerases (G:C and A:T mutations) as well as a propensity to develop tandem point mutations.
4) The fish Ig mutator AID is found to be fully functional for SHM and CSR though enzyme kinetics vary with species and temperature. Furthermore all regulatory aspects of fish AID expression and sub-cellular localization studied to date are also functionally conserved with AID from homeotherms.
5) Recent evidence from sharks indicates that a form of CSR (class switch recombination) developed among the earliest divergent gnathostomes.
6) In early gnathostomes the actual protein affinities are not raised to the extent seen in homeotherms though this may relate either to differences in affinity maturation kinetics or in the manner by which cells undergo selection. This remains one of the outstanding issues to be resolved.
